# The vascular protective effect of matrix Gla protein during kidney injury

**DOI:** 10.3389/fmmed.2022.970744

**Published:** 2022-11-01

**Authors:** Yujiro Kida, Ikuyo Yamaguchi

**Affiliations:** ^1^ Center for Tissue and Cell Sciences, Seattle Children’s Research Institute, Seattle, WA, United States; ^2^ Department of Nephrology, Takashimadaira Chūō General Hospital, Tokyo, Japan; ^3^ Division of Pediatric Nephrology and Hypertension, Department of Pediatrics, The University of Oklahoma Health Sciences Center, Oklahoma Children’s Hospital, OU Health, Oklahoma City, OK, United States

**Keywords:** matrix Gla protein, angiogenesis, vascular endothelial growth factor-A (VEGF-A), peritubular capillaries, endothelial cells, pericytes, vascular damage, kidney injury

## Abstract

Matrix Gla protein (MGP) is a small secreted protein and requires vitamin K dependent γ-carboxylation for its function. MGP has been identified as a local inhibitor of vascular calcification because MGP-deficient mice die due to severe arterial calcification and resulting arterial rupture. Clinical trials revealed that reduction in active MGP predicts poor prognosis in patients due to cardiovascular complications. However, recent studies showed that MGP controls angiogenesis during development. MGP-deficient mice demonstrated abnormal hypervascularization and arteriovenous malformations in kidneys and other organs. This abnormal angiogenesis is largely caused by excessive expression of vascular endothelial growth factor-A (VEGF-A) and VEGF receptor-2 (VEGFR2). However, only a few studies have investigated the roles of MGP in tissue injury. We observed mesangial cell proliferation and mild interstitial fibrosis in addition to increased capillaries in kidneys of MGP-null mice even without injury. We also created a mouse model with kidney injury and found that kidney damage greatly increases MGP expression in peritubular capillary endothelial cells and tubular epithelial cells. Finally, our study showed that impairment of MGP expression aggravates peritubular capillary rarefaction and accumulation of collagen-producing myofibroblasts following kidney injury. Peritubular capillary damage induces capillary loss as well as trans-differentiation of vascular pericytes into myofibroblasts. These results indicate that MGP has the vascular protective effect in the injured kidney. Clinical trials have already started to test the efficacy of MGP activation to repair vascular calcification in patients with chronic kidney diseases. In this “Hypothesis and Theory” article, we discuss possible mechanisms by which MGP protects against vascular damage during tissue injury based on our experimental results and previous results from other research groups.

## Introduction

Blood vessels are a complex network of tubes that transport oxygenated blood and nutrients throughout our bodies. If laid end to end, these vessels including arteries, veins, and capillaries from one typical adult would circle the Earth twice. The growth of blood vessels is essential for organ development and tissue repair (Carmeliet and Jain, 2011). Our bodies keep a tight balance between proangiogenic factors (induction of angiogenesis) and antiangiogenic factors (inhibition of angiogenesis) to prevent unnecessary vessel formation. An imbalance of this angiogenic process has a major impact on our health and contributes to the pathogenesis of various diseases including cancer, ischemic disorders and tissue fibrosis (Carmeliet, 2003). In the kidney, both excessive and impaired angiogenesis cause disruption of normal kidney architecture without any injurious insults (Liu et al., 2007; Hakroush et al., 2009; Dimke et al., 2015). Therefore, there is a great need for a better understanding of angiogenic processes in normal and diseased organs to develop novel therapies. Recent studies have revealed that matrix Gla protein (MGP) is a critical regulator of angiogenesis in multiple organs including the lung and the kidney during development (Yao et al., 2011). MGP has been identified as a potent inhibitor of vascular calcification (Luo et al., 1997). Vascular calcification is a process characterized by thickening and loss of elasticity of muscular artery walls. Importantly, vascular calcification is common and significantly associated with increased morbidity and mortality in patients with chronic kidney disease (CKD) (Demer and Tintut, 2008; Shroff et al., 2013). In this “Hypothesis and Theory” article, we describe 1) the activation process of MGP, 2) inhibitory roles of MGP in vascular calcification, 3) roles of MGP in normal angiogenesis, 4) roles of MGP in angiogenesis post-injury, and 5) therapeutic implications of MGP activation.

## Structure and activation of MGP

**FIGURE 1:**
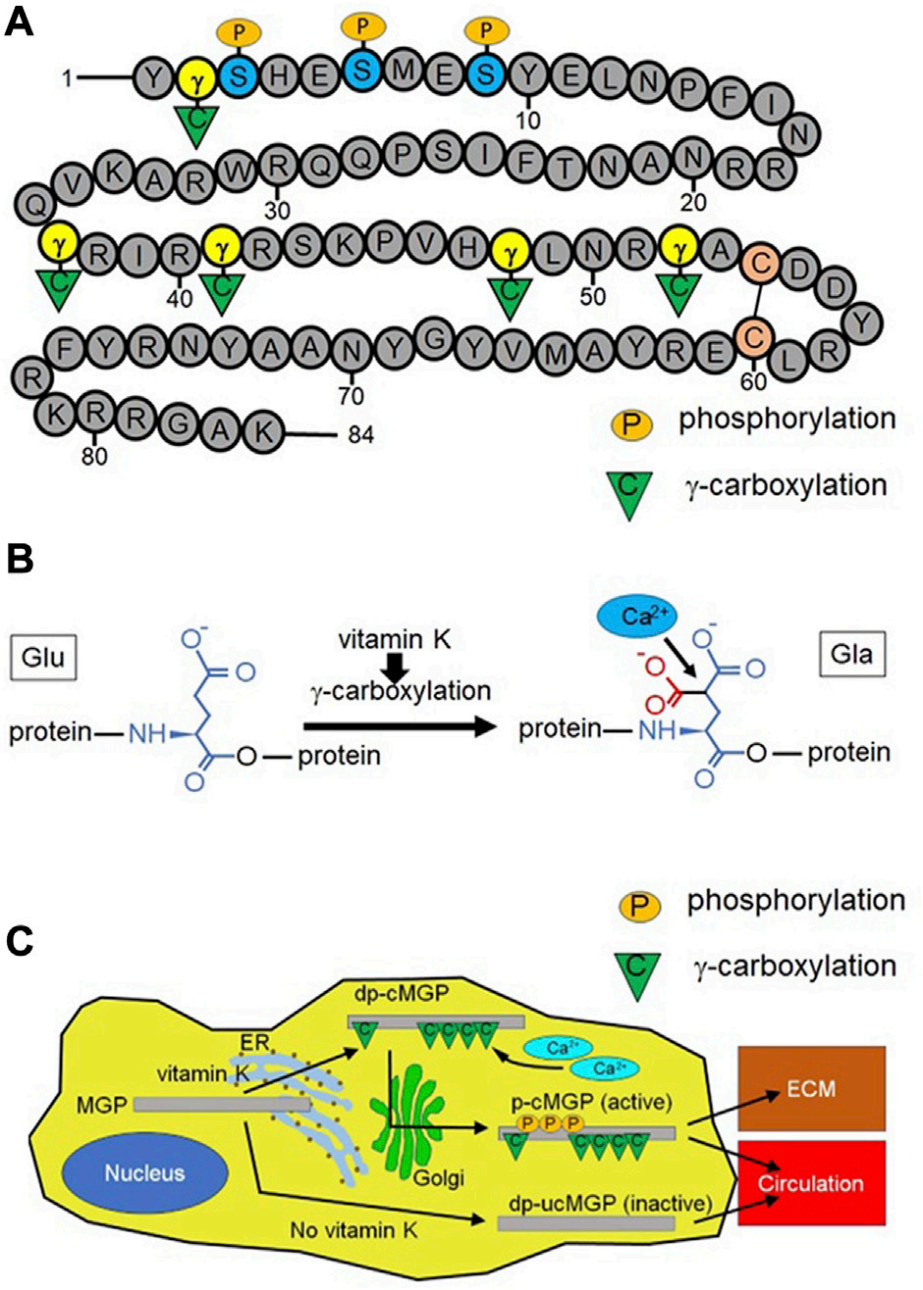
Structure, calcium binding function, and activation process of matrix Gla protein (MGP). **(A)** MGP is composed of 84 amino acids and contains three serine residues that could be phosphorylated by a Golgi-casein kinase (at positions 3, 6, and 9) and five glutamate residues that could be γ-carboxylated (at positions 2, 37, 41, 48, and 52). These two types of posttranslational modification (serine-phosphorylation and γ-glutamate carboxylation) activate MGP. MGP has a single disulfide bond between two cysteine residues (at positions 54 and 60). Number indicates the position of each amino acid. γ: γ-glutamate, S: serine, C in the circle: cysteine, P in the orange ellipse: phosphorylation, C in the green triangle: γ-carboxylation **(B)** Vitamin K-dependent γ-carboxylation converts glutamate (Glu) into γ-carboxyglutamate (γ-carboxyglutamic acid) (Gla). This reaction increases the negative charge of MGP, promoting electrical binding between MGP and positively charged calcium ions. A red portion is newly attached to Glu after the γ-carboxylation reaction. **(C)** Naïve MGP undergoes γ-carboxylation in the endoplasmic reticulum (ER) in a vitamin K dependent manner, resulting in dephosphorylated (desphosphorylated) carboxylated MGP (dp-cMGP). Subsequently, dp-cMGP undergoes serine-phosphorylation by Golgi-casein kinase in the Golgi apparatus (Golgi). The resulting phosphorylated carboxylated MGP (p-cMGP) is the fully active form, which is largely located at the extracellular matrix (ECM). A small portion of p-cMGP moves into the circulation. Under vitamin K deficiency, MGP is neither phosphorylated nor γ-carboxylated (dephosphorylated uncarboxylated MGP, dp-ucMGP). This inactive form of MGP also moves into the circulation.

MGP is a small secreted protein (10.6 kD in human) that contains multiple γ-carboxyglutamate (Gla) amino-acid residues and one disulfide bond (Figure 1A) (Price et al., 1983; Hackeng et al., 2001). MGP is activated by two posttranslational modifications, serine phosphorylation and vitamin-K dependent γ-glutamate carboxylation (Figures 1A–C) (Hackeng et al., 2001; Schurgers et al., 2007). After active MGP is released into the extracellular space, it is mainly localized at the extracellular matrix (ECM) and regulates vascular homeostasis.

## Inhibitory roles of MGP in vascular calcification

MGP has been identified as a local inhibitor of calcification, because MGP-deficient mice die within 2 months due to severe arterial calcification and following arterial rupture without atherosclerotic plaques (Luo et al., 1997; Malhotra et al., 2019). Consistently, arterial calcification is detected in some of patients with Keutel syndromes where loss-of-function mutations in the MGP gene are identified (Cancela et al., 2021). Vascular calcification occurs in two distinct sites, intimal and medial layers of arterial walls. While intimal calcification is related to atherosclerotic plaques, medial calcification, also known as Mönckeberg’s sclerosis, is characterized by vascular stiffening and arteriosclerosis (Shroff et al., 2013). As MGP-deficient mice demonstrate arterial calcification without evidence of atherosclerotic plaques (Luo et al., 1997), impairment of MGP function mainly contributes to medial calcification and vascular stiffening. Medial calcification is commonly detected in patients with CKD (Shroff et al., 2013).

### MGP suppresses vascular calcification *via* multiple pathways

MGP is composed of 84 amino acids and contains three serine residues that could be phosphorylated (at positions 3, 6, and 9) and five glutamate residues that could be γ-carboxylated (at positions 2, 37, 41, 48, and 52) (Figure 1A). Full length of MGP with γ-carboxylation (amino acids 1–84), γ-carboxylated MGP fragment (amino acids 35–54), and serine phosphorylated MGP fragment (amino acids 3–15) inhibit calcification whereas MGP fragment (amino acids 54–84), uncarboxylated MGP fragment (amino acids 35–54), and dephosphorylated MGP fragment (amino acids 3–15) have no effect on calcification (Schurgers et al., 2007). This result indicates that posttranslational modification (γ-carboxylation or serine phosphorylation) is necessary for MGP to inhibit tissue calcification. γ-carboxylation of glutamate residues increases the negative charge of MGP, which facilitates binding between MGP and positively charged calcium ions (Figure 1B). This function of MGP inhibits incorporation of calcium ions into hydroxyapatite (the basic component of vascular calcification) (Nigwekar et al., 2018). Separately, MGP directly interacts with growing hydroxyapatite crystals and absorbs them (O’Young et al., 2011). MGP without serine phosphorylation or γ-glutamate carboxylation markedly reduces ability to disassemble hydroxyapatite. Fetuin-A (α_2_-Heremans-Schmid glycoprotein) is a negatively charged protein, mostly released by the liver, which scavenges calcium ions and phosphate ions from the serum (Ketteler et al., 2003; Schäfer et al., 2003). The interaction between fetuin-A and γ-carboxylated MGP integrates calcium and phosphate clusters into amorphous proteinaceous spherical particles called calciprotein particles, 50–500 nm in diameter (Kutikhin et al., 2021). These particles transport calcium and phosphate to bones and prevent vascular calcification by inhibiting growth and aggregation of hydroxyapatite (Jahnen-Dechent et al., 2011). MGP deficiency upregulates the expression of runt-related transcription factor 2 (Runx2), which promotes trans-differentiation of vascular smooth muscle cells (VSMCs) into osteochondrogenic cells (Malhotra et al., 2022). These transdifferentiated VSMCs secrete calcium ions and phosphate ions, accelerating vascular calcification (Speer et al., 2009; Neven et al., 2011). Bone morphogenetic protein-2 (BMP-2) is one of major ligands that induce osteochondrogenic VSMC by upregulation of Runx2 (Lee et al., 2003; Li et al., 2008; Nakagawa et al., 2010). MGP binds to BMP-2, sequestering it from its receptor (Zebboudj et al., 2002; Zebboudj et al., 2003). Moreover, MGP-deficient VSMCs disrupt elastin fiber integrity and enhance collagen synthesis in the media of arteries, further increasing vascular stiffness (Malhotra et al., 2022). In MGP-deficient mice, raising the serum level of MGP does not affect vascular or ECM calcification, indicating that MGP regulates tissue calcification locally but not systemically (Murshed et al., 2004).

### Clinical evidences for inhibitory effect of MGP on vascular calcification

Calciphylaxis is a rare and life-threatening disease where severe vascular calcification appears in the fat and skin tissues. This disorder typically occurs in patients with CKD (Nigwekar et al., 2018). Although patients with calciphylaxis increase expression levels of uncarboxylated MGP and γ-carboxylated MGP in affected tissues, uncarboxylated MGP expression is predominantly upregulated compared to γ-carboxylated MGP expression (Kramann et al., 2013). Thus, those patients reduce relative γ-carboxylated MGP expression (calculated by [γ-carboxylated MGP expression]/[total MGP expression]) in affected tissues (Kramann et al., 2013; Nigwekar et al., 2017; Nigwekar et al., 2018), supporting the anti-calcification function of active MGP in patients. The circulating level of inactive MGP (dephosphorylated and uncarboxylated MGP, dp-ucMGP) is related to the severity of aortic calcification in patients with CKD (Schurgers et al., 2010), and predicts long-term mortality in patients with calcific valvular aortic stenosis (Ueland et al., 2010). Higher circulating inactive MGP is also associated with higher risk of heart failure and peripheral artery disease (Dalmeijer et al., 2013; Malhotra et al., 2022). Excessive arterial stiffness due to impaired MGP activation increases the pulsatile afterloads for the left ventricle and reduces coronary artery perfusion pressure during diastole, leading to heart failure (Chirinos, 2022). These results imply that activated MGP protects against vascular calcification and resulting cardiovascular diseases.

## Roles of MGP in normal angiogenesis

### Abnormal angiogenesis in MGP-null mice

In MGP-deficient mice, the calcification affects all elastic and muscular arteries, but not arterioles, veins, or capillaries (Luo et al., 1997). This finding suggests that a major role of MGP in capillaries is not inhibition of calcification, but is related to angiogenesis. Yao et al. find that MGP-deficient mice have abnormal vasculature and arteriovenous malformations in lungs and kidneys (Yao et al., 2011). MGP-null mice showed more vascularized lungs and kidneys than wild-type control mice, whereas transgenic mice with MGP overexpression (MGP transgenic mice) exhibited less vascularized lungs and kidneys than wild-type mice (Yao et al., 2011). In the kidneys from MGP-null mice, the number of glomeruli per microscopic field is increased (3 times more than wild-type), and peritubular capillary density is also increased (4 times more than wild-type). This enhanced angiogenesis detected in MGP knockout mice creates dysfunctional blood vessels because abnormal arteriovenous shunting frequently appears, allowing the blood to bypass glomerular and peritubular capillaries (Yao et al., 2011). Increased microvascular density is also reported in cardiac muscles and skeletal muscles of MGP knockout mice (Sharma and Albig, 2013).

### Excessive expression of vascular endothelial growth factor-A in MGP-deficient kidneys

What are the mechanisms underlying abnormal angiogenesis in MGP-deficient kidneys? The leading explanation is increased expression of vascular endothelial growth factor-A (VEGF-A) in MGP-null kidneys (6 times more than in control kidneys from wild-type mice) (Yao et al., 2013). Excessive VEGF-A induces abnormal angiogenesis and disorganizes glomerular and peritubular capillary structure (Liu et al., 2007; Hakroush et al., 2009). In addition to unusually high expression of VEGF-A, expression of activin receptor-like kinase receptor 5 (ALK5) is also upregulated in MGP knockout kidneys (Yao et al., 2011). ALK5 is a transforming growth factor-β (TGF-β) type I receptor. Upon activation by TGF-β, abundantly expressed ALK5 on tubular epithelial cells enhances protein expression of hypoxia-inducible factor-1α (HIF-1α) (Basu et al., 2011), which triggers VEGF-A synthesis in those cells (Manotham et al., 2005; Kimura et al., 2008). Tubular epithelial cells (in the thick ascending limbs of Henle’s loop, distal tubules, and proximal tubules) and podocytes are major sources of VEGF-A in the kidney (Dimke et al., 2015). It is not clear whether glomerular abnormality in MGP-null mice mainly arises from increased tubular VEGF-A expression or increased podocyte VEGF-A expression, because there is no information about expression of ALK5 and VEGF-A in podocytes from MGP knockout mice. There is clear evidence that VEGF-A is involved in bone formation and osteoblast differentiation (Maes et al., 2002; Street et al., 2002). Given the similarities between bone formation and vascular calcification, VEGF-A may play a role in vascular calcification (Schroff et al., 2013). Indeed, VEGF-A could upregulate BMP-2 expression in cultured endothelial cells (Bouletreau et al., 2002). Therefore, increased VEGF-A expression could damage arteries by calcium deposition in MGP knockout mice.

### Intrinsic abnormality of MGP-deficient endothelial cells in kidneys

MGP knockout mice also increase expression of VEGF receptor-2 (VEGFR2) in the kidney (15 times more than wild-type) (Yao et al., 2016). Although VEGFR2 is predominantly expressed in endothelial cells in normal kidneys (Schrijvers et al., 2004), this increase in VEGFR2 expression (15 times more than wild-type) cannot be explained by the increase in glomerular and peritubular capillary density (3–4 times more than wild-type) in MGP-null kidneys. Thus, loss of endothelial MGP seems to upregulate VEGFR2 expression in endothelial cells. The long-term and intense stimulation of VEGFR2 is sufficient to induce abnormal capillary proliferation and glomerular sclerosis in mice without development of interstitial fibrosis (Sato et al., 2011). Furthermore, increased VEGFR2 expression is typically observed in the early stage of diabetic nephropathy, where mesangial matrix deposition is prominent (Cooper et al., 1999). Sharma and Albig report that MGP-null aortic rings enhance vessel sprouting compared to aortic rings from wild-type mice (Sharma and Albig, 2013). Although MGP-deficient aortic rings are reported to upregulate Notch signaling activity compared to wild-type aortic rings (Sharma and Albig, 2013), Notch activation might be secondary to excessive sprouting of MGP-deficient endothelial cells, because Notch signaling restricts disorganized sprouting and branching of endothelial cells (Hellström et al., 2007; Kida et al., 2016). Anti-sense-mediated knockdown of MGP in zebrafish embryos results in no blood flow in some of the major vessels (Sharma and Albig, 2013). Overall, these findings confirm that MGP-null endothelial cells also contribute to abnormal vessel formation.

### Spontaneous damage of normal kidney structure in MGP-deficient mice

**FIGURE 2:**
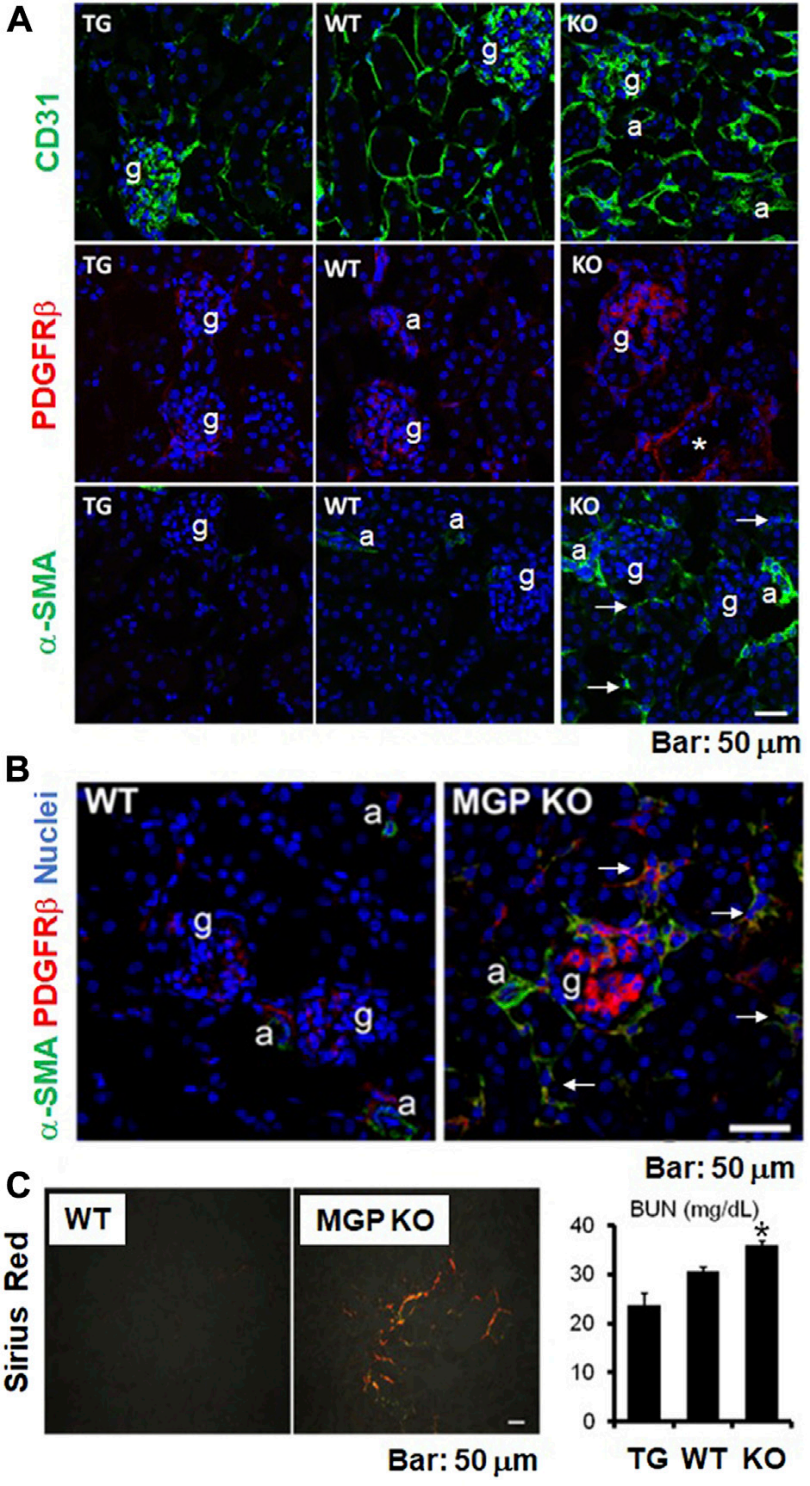
Structural and functional abnormalities in kidneys of MGP-null mice. **(A)** Immunofluorescent staining for CD31, platelet derived growth factor receptor-β (PDGFRβ), and α-smooth muscle actin (α-SMA) in uninjured kidneys of MGP transgenic, wild-type, and MGP knockout mice. Staining for CD31 (green, endothelial marker) revealed that peritubular capillary (PTC) density is increased in MGP knockout mice, whereas PTC density is lowered in MGP transgenic mice compared with wild-type mice (top panel). Staining for PDGFRβ (red) demonstrated that PDGFRβ^+^ mesangial cells in the glomerulus proliferate in MGP knockout mice (middle panel). Staining for α-SMA (green, myofibroblast marker) identified the emergence of α-SMA^+^ myofibroblasts in MGP knockout mice (bottom panel). Arrows indicate α-SMA^+^ myofibroblasts in the interstitium of MGP-null kidneys without injury. Note that α-SMA expression is detectable in arterioles and small arteries, but not in interstitium of normal kidneys. TG: MGP transgenic mice, WT: wild-type mice, KO: MGP knockout mice, g: glomerulus, a: arteriole, asterisk: small artery, blue: nuclear staining. Scale bar, 50 μm. **(B)** Double immunofluorescent staining for PDGFRβ (red) and α-SMA (green) using uninjured kidneys of wild-type and MGP knockout mice. Compared with wild-type mice, PDGFRβ^+^ mesangial cells in the glomerulus and PDGFRβ^+^ pericytes in the interstitium proliferate in MGP-deficient mice. Most of the PDGFRβ^+^ pericytes in the interstitium are positive for α-SMA in MGP-null kidneys (indicated by arrows), demonstrating that MGP deficiency induces pericyte-myofibroblast transition. WT: wild-type mice, MGP KO: MGP knockout mice, g: glomerulus, a: arteriole, blue: nuclear staining. Scale bar, 50 μm. **(C)** Picrosirius red-stained fibrillar collagens in uninjured kidneys of wild-type and MGP knockout mice (left panel). Fibrillar collagen deposition detected by a polarized light increased in MGP-null kidneys. WT: wild-type mice, MGP KO: MGP knockout mice, Scale bar, 50 μm. Measurement of blood urea nitrogen (BUN) in mice without injury (right panel). BUN levels were elevated in MGP knockout mice compared with wild-type mice, suggesting that MGP deficiency induces impaired glomerular filtration ratio (GFR). TG: MGP transgenic mice, WT: wild-type mice, KO: MGP knockout mice. Results are presented as mean ± SEM. *n* = 8–9/group. *p < 0.05 versus wild-type mice by Student’s *t*-test.

**FIGURE 3:**
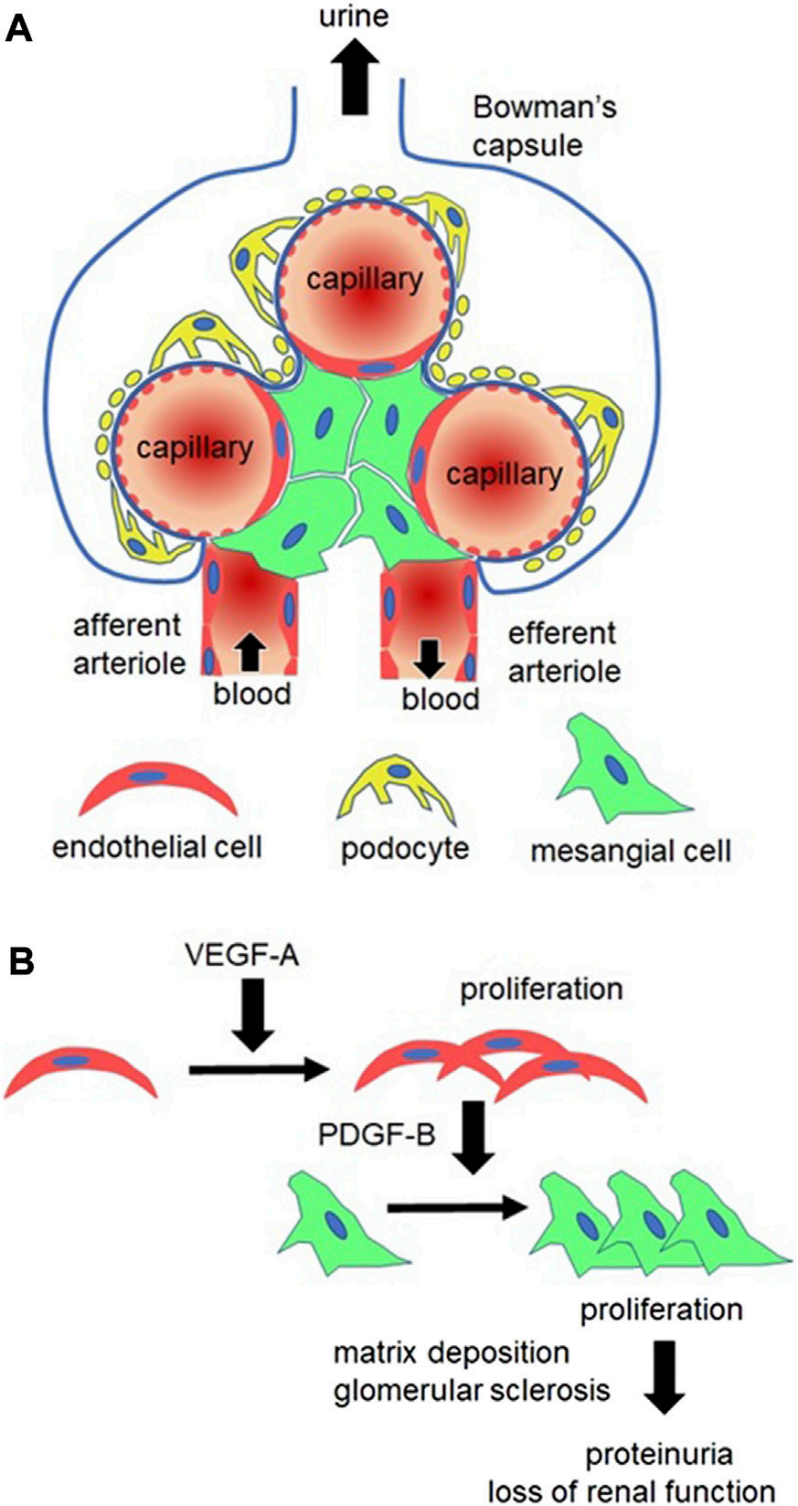
Glomerular alterations in MGP-deficient mice. **(A)** Structure of the healthy glomerulus. Glomerular capillaries are supported by two types of cells, podocytes (yellow) and mesangial cells (green). Podocytes nurture capillary endothelial cells (red) by synthesizing vascular endothelial growth factor-A (VEGF-A). Podocyte foot processes and capillary basement membranes (indicated by bold blue lines) constitute capillary barriers to prevent proteinuria (plasma protein leakage into urine). Mesangial cells are contractile cells expressing platelet derived growth factor receptor-β (PDGFRβ). They surround capillaries to control capillary flow, determining the single-nephron glomerular filtration ratio (GFR). Capillary blood flows into the glomerulus *via* an afferent arteriole and flows out of the glomerulus *via* an efferent arteriole. **(B)** In MGP-deficient kidneys, glomerular endothelial cells (red) remarkably proliferate due to increased VEGF-A expression. In response to VEGF-A stimulation, endothelial cells synthesize PDGF-B, leading to proliferation of PDGFRβ^+^ mesangial cells (green). Mesangial cells form mesangial matrix and compress capillary tubes, leading to disruption of capillary basement membrane and capillary occlusion. This ultimately causes proteinuria and loss of the whole-kidney GFR (loss of kidney function).

We investigated the renal vascular phenotypes of MGP-deficient mice and MGP transgenic mice. As reported previously, MGP-null kidneys demonstrate increased peritubular capillary density, whereas MGP transgenic kidneys show reduced density (Figure 2A, our unpublished data, see details in Supplementary Material). We also observe that platelet derived growth factor receptor-β (PDGFRβ) positive cells are increased in the glomeruli and in the interstitium of MGP-deficient kidneys (Figures 2A,B, our unpublished data, see details in Supplementary Material), indicating that intraglomerular PDGFRβ^+^ mesangial cells and interstitial PDGFRβ^+^ microvascular pericytes are activated and proliferate in the absence of any external injury. MGP-deficient kidneys also have increased tissue fibrosis and collagen-producing myofibroblasts that are positive for α-smooth muscle actin (α-SMA) (Figures 2A–C, our unpublished data, see details in Supplementary Material). Renal function is also slightly but significantly impaired in MGP knockout mice compared with wild-type control mice (Figure 2C, our unpublished data, see details in Supplementary Material). These characteristics are reminiscent of vascular formation in VEGF-A overexpressing kidneys (Liu et al., 2007; Hakroush et al., 2009). In response to overexpression of VEGF-A, glomerular and peritubular capillary endothelial cells strongly express PDGF-B and TGF-β (Hakroush et al., 2009). In the glomeruli, enhanced PDGF-B expression stimulates mesangial cell proliferation and mesangial matrix deposition, leading to glomerular sclerosis (Johnson et al., 1992) (Figures 3A,B). In the interstitial spaces, PDGF-B causes proliferation of PDGFRβ^+^ pericytes and TGF-β causes differentiation of PDGFRβ^+^ pericytes into α-SMA^+^ myofibroblasts, respectively (Wu et al., 2013). High levels of VEGF-A are sufficient to produce glomerular sclerosis, proteinuria, and impaired kidney function over the long term (Liu et al., 2007). Although the glomerular size is considerably increased in kidneys with VEGF-A overexpression (Liu et al., 2007; Hakroush et al., 2009), we do not see enlarged glomeruli in MGP-null kidneys (Figure 2A, our unpublished data, see details in Supplementary Methods), suggesting that some of the abnormal findings in MGP-deficient kidneys are not explained well by enhanced expression of VEGF-A. In combination, our observations and these previous results suggest that robust expression of VEGF-A and VEGFR2 and resultant immature vessel formation in MGP-null mice are sufficient to initiate kidney injury without any injurious stimulation.

### MGP may not affect Bone morphogenetic protein-2 or BMP-4 signaling in peritubular capillary endothelial cells

**FIGURE 4:**
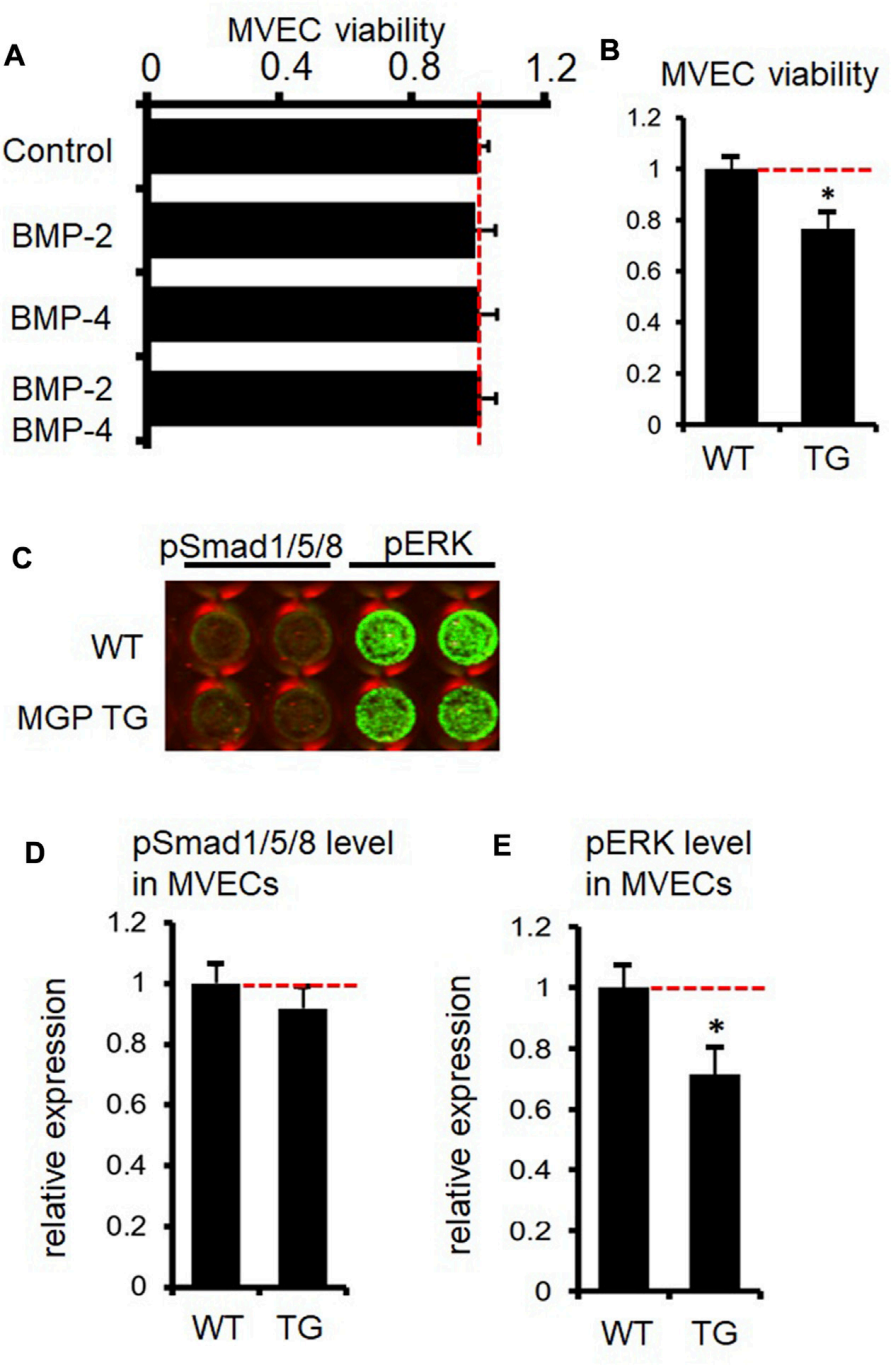
Absence of inhibitory effect of MGP on BMP-signaling in kidney capillary endothelial cells. **(A)** Proliferation of microvascular endothelial cells (MVECs) of peritubular capillaries was assessed by MTS assay after MVECs were incubated with BMP-2 or BMP-4 for 3 days. Neither BMP-2, BMP-4 nor BMP-2/BMP-4 had a significant effect on proliferation of kidney MVECs. Results are presented as mean ± SEM. *n* = 4/group. **(B)** Results of MTS assay showed that proliferation of MVECs from MGP transgenic (TG) kidneys is less than that of MVECs from wild-type (WT) kidneys. Results are presented as mean ± SEM. *n* = 4/group. *p < 0.05 versus MGP WT MVECs by Student’s *t*-test. **(C)** Representative image of the signal intensity of phosphorylated Smad1/5/8 (pSmad1/5/8) and phosphorylated ERK (pERK) in MVECs isolated from wild-type (WT) and MGP transgenic (MGP TG) kidneys assessed by in-cell Western assay. The intensity of green color in each well indicates the protein expression level. Note almost no expression of pSmad1/5/8 both in WT and TG MVECs. **(D)** Quantification of pSmad1/5/8 signal intensity in MVECs isolated from wild-type (WT) and MGP transgenic (TG) kidneys. There was no significant difference in pSmad1/5/8 expression between WT and TG MVECs. Results are presented as mean ± SEM. *n* = 4/group. **(E)** Quantification of pERK signal intensity in MVECs isolated from wild-type (WT) and MGP transgenic (TG) kidneys. pERK expression was impaired in TG MVECs compared to WT MVECs. Results are presented as mean ± SEM. *n* = 4/group. *p < 0.05 versus MGP WT MVECs by Student’s *t*-test. Red dotted lines indicate 1.0 (reference value in the control group) in Figures 4A,B,D,E.

Previous studies demonstrate that MGP suppresses proliferation of bovine and human aortic endothelial cells *via* inhibition of BMP signaling (Yao et al., 2009; Yao et al., 2011). Therefore, we investigated whether MGP inhibits BMP signaling in peritubular capillary endothelial cells. We incubate peritubular capillary endothelial cells with BMP-2, BMP-4, or a combination of BMP-2 and BMP-4 for 3 days. Peritubular capillary endothelial cells are isolated from the kidneys of wild-type mice by using fluorescence-activated cell sorting (FACS) as we previously reported (Kida et al., 2013) (See details in Supplementary Material). However, neither BMP-2 nor BMP-4 affect proliferation of wild-type peritubular capillary endothelial cells as assessed by MTS assay (Figure 4A, our unpublished data, see details in Supplementary Material). Peritubular capillary endothelial cells isolated from MGP transgenic kidneys are significantly less proliferative than those from wild-type control kidneys (Figure 4B, our unpublished data, see details in Supplementary Material), which is consistent with our *in vivo* results of reduced capillary density in MGP transgenic kidneys (Figure 2A). By using in-cell Western assay, we examine whether MGP suppresses expression levels of phosphorylated Smad1/5/8 (a canonical downstream target of BMP-2 or BMP-4) in peritubular capillary endothelial cells. There is no difference in phosphorylated Smad1/5/8 expression between control wild-type and MGP transgenic endothelial cells (Figures 4C,D, our unpublished data, see details in Supplementary Material). Moreover, we detect almost no activation of Smad1/5/8 in wild-type endothelial cells (Figure 4C). Instead, we find that overexpression of MGP downregulates expression of phosphorylated ERK (Figure 4E, our unpublished data, see details in Supplementary Material), which is consistent with reduction in VEGFR2 activity. Activated ERK is a major downstream target of VEGFR2 signaling to induce endothelial cell proliferation (Kroll and Waltenberger, 1997). Although BMP-4 could activate ERK in human umbilical vein endothelial cells, ERK activation with BMP-4 accompanies phosphorylation of Smad1/5/8 (Zhou et al., 2007). Collectively, we do not confirm that MGP inhibits BMP-2 or BMP-4 signaling in peritubular capillary endothelial cells. This discrepancy could be explained by different response to BMP stimulation between aortic endothelial cells and peritubular capillary endothelial cells, because microvascular endothelial cells within different tissues possess distinct phenotypes (Nolan et al., 2013).

## Roles of MGP in vascular damage in the kidney

### Increased expression of total MGP following kidney injury

As we described above, MGP is essential for physiological vascular growth and maintenance (normal angiogenesis). Does MGP also play a role in vascular damage during tissue injury? As far as we know, only a few studies have investigated how MGP works following tissue injury. In atherosclerotic mouse models, MGP overexpression ameliorates atherosclerosis and arterial calcification (Yao et al., 2010), suggesting that active MGP protects against vascular injury. Although total MGP expression is significantly enhanced in the kidney in subtotal nephrectomy models and renal ischemia/reperfusion injury models (Basile et al., 2005; Miyata et al., 2018), we still do not know whether MGP counteracts kidney injury.

### Enhancement of kidney injury due to impaired MGP expression

**FIGURE 5:**
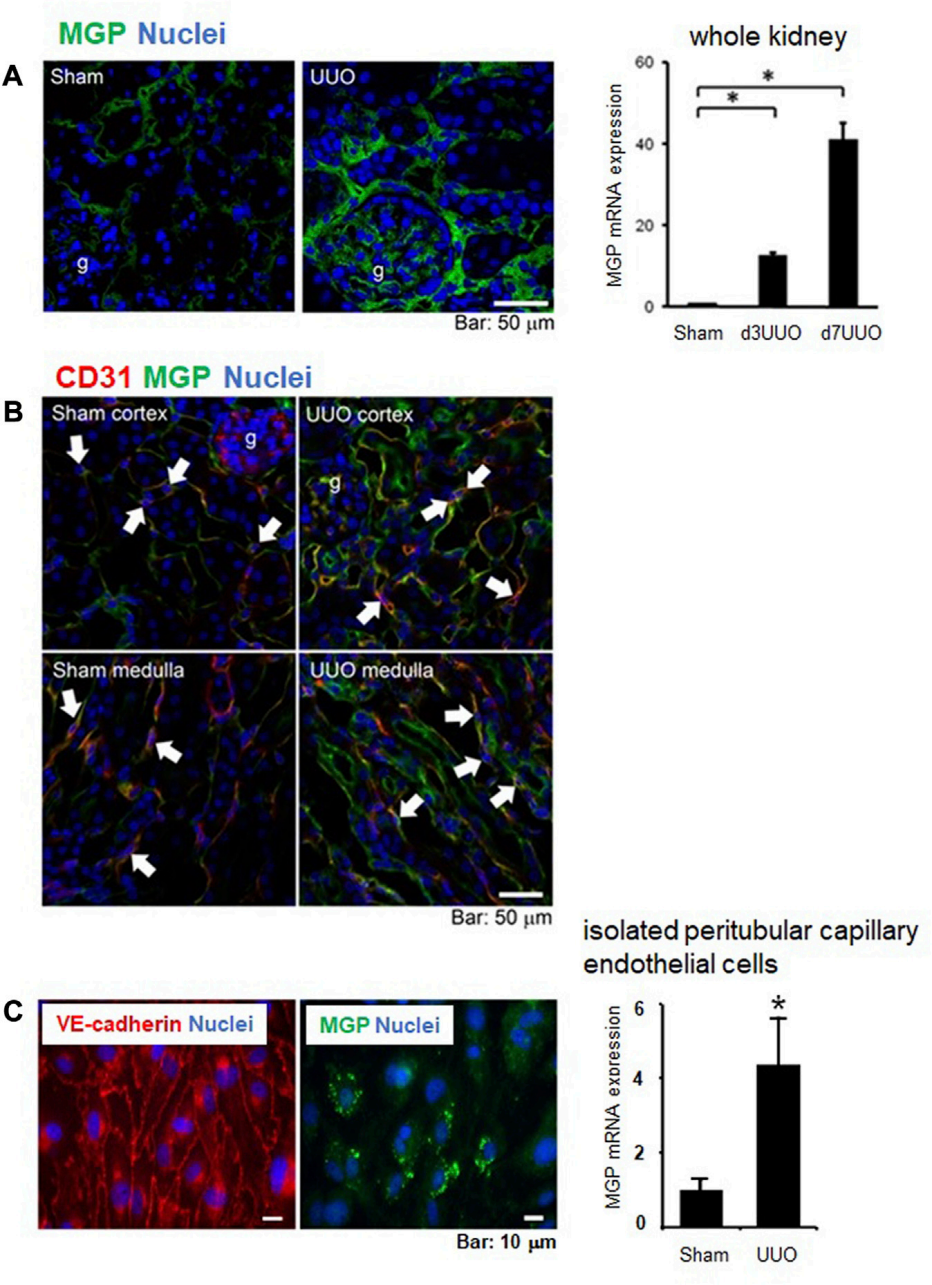
MGP expression in kidneys of wild-type mice following injury. **(A)** Immunofluorescent staining for MGP (green) in control (Sham surgery) and UUO kidneys (7 days post-UUO surgery) (left panel). MGP expression was increased in glomerular cells, interstitial cells, and tubular epithelial cells after UUO injury. g: glomerulus. blue: nuclear staining. Scale bar, 50 μm. *Mgp* transcripts in the whole kidneys were also increased along the time course after UUO injury (right panel). d3UUO: 3 days post-UUO, d7UUO: 7 days post-UUO. Results are presented as mean ± SEM. *n* = 5–6/group. *p < 0.05 versus sham group by Student’s *t*-test. **(B)** MGP was expressed in glomerular and capillary endothelial cells in addition to tubular epithelial cells in the cortex and the medulla of control (Sham surgery) and UUO kidneys (day 3 post-UUO surgery). UUO kidney injury enhanced MGP expression in glomerular and peritubular capillaries as well as the apical side of tubules. Arrows indicate peritubular capillary endothelial cells positive for MGP (green) and CD31 (red, endothelial marker). g: glomerulus, blue: nuclear staining. Scale bar, 50 μm. **(C)** Peritubular capillary endothelial cells were isolated by fluorescence-activated cell sorting (FACS). FACS separated a cell population positive for CD31 (endothelial marker), and negative for CD11b and CD45 (leukocyte marker). Isolated endothelial cells were cultured and stained for VE-cadherin (red, endothelial marker) and MGP (green) (left panel). blue: nuclear staining. Scale bar, 10 μm. *Mgp* transcripts were measured in isolated peritubular capillary endothelial cells (right panel). *Mgp* mRNA was increased in capillary endothelial cells from UUO kidneys compared to those from control (Sham surgery) kidneys. UUO: 3 days post-UUO. Results are presented as mean ± SD. *n* = 5 for sham, *n* = 6 for UUO group. *p < 0.05 versus sham group by Student’s *t*-test.

**FIGURE 6:**
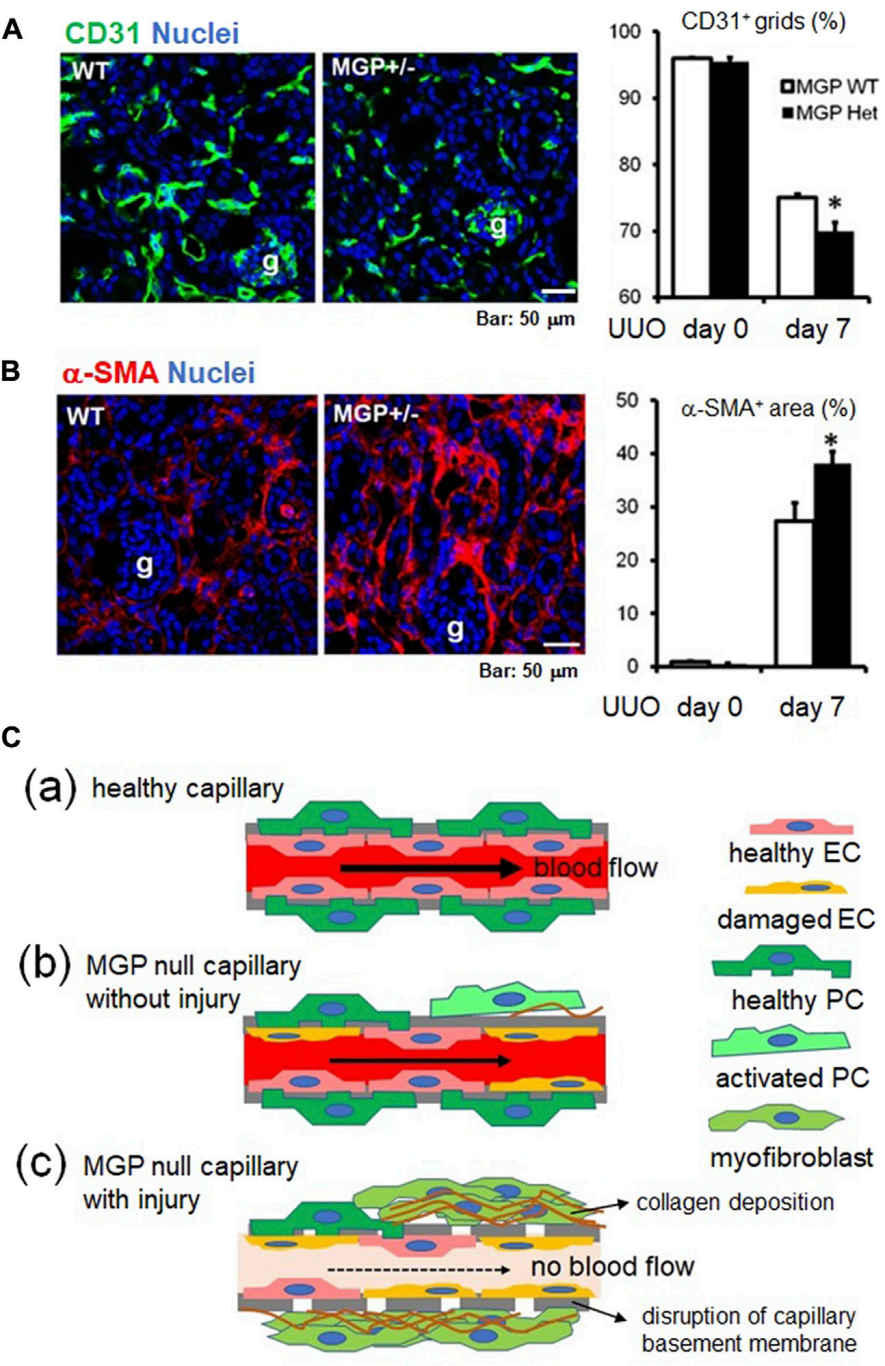
The vascular protective effect of MGP during kidney injury. **(A)** Immunofluorescent staining for CD31 (green, endothelial marker) using kidneys of wild-type and MGP^+/−^ mice (left panel). Remarkable capillary regression was detected in MGP^+/−^ mice 7 days after UUO compared with wild-type mice. g: glomerulus. Peritubular capillary (PTC) density was assessed by counting the percentage of CD31^+^ grids (right panel). PTC density was significantly decreased in MGP^+/−^ mice compared with wild-type mice 7 days post-UUO injury. WT: wild-type, MGP^+/−^: MGP heterozygous knockout mice. Results are presented as mean ± SEM. *n* = 4/group. *p < 0.05 versus MGP WT group by Student’s *t*-test. g: glomerulus, Scale bar, 50 μm. **(B)** Immunofluorescent staining for α-smooth muscle actin (α-SMA) (red, myofibroblast marker) using kidneys of wild-type and MGP^+/−^ mice (left panel). Remarkable myofibroblast accumulation was detected in MGP^+/−^ mice 7 days after UUO compared with wild-type mice. Myofibroblast accumulation was quantified by measuring the percentage of α-SMA^+^ area (right panel). α-SMA^+^ area was significantly enlarged in MGP^+/−^ mice compared with wild-type mice 7 days post-UUO injury. WT: wild-type, MGP^+/−^: MGP heterozygous knockout mice. Results are presented as mean ± SEM. *n* = 4/group. *p < 0.05 versus MGP WT group by Student’s *t*-test. g: glomerulus, Scale bar, 50 μm. **(C)** Schematic showing capillary damage in MGP-null mice with or without injury. **(C-a)** In a healthy capillary, capillary endothelial cells (healthy EC, pink) are lining capillary basement membranes (indicated by bold gray lines) and are closely attached to pericytes (healthy PC, green). **(C-b)** In MGP-null capillaries without injury, some capillary endothelial cells are spontaneously damaged. Damaged endothelial cells (damaged EC, orange) activate some pericytes. Activated pericytes (activated PC, light green) migrate away from capillary endothelial cells and start to produce the extracellular matrix components such as collagen (indicated by brown wavy lines). Activated endothelial cells induce platelet aggregation, leading to coagulation. This impairs the capillary blood flow. **(C-c)** In MGP-null capillaries with injury, capillary endothelial cells are further activated and damaged. Activated pericytes transdifferentiate into collagen-producing myofibroblasts (moss green), leading to tissue fibrosis. Without support by pericytes, capillary basement membrane is disrupted, resulting in the capillary occlusion with no blood flow.

To characterize roles of MGP in vascular damage following kidney injury, we subjected wild-type mice to unilateral ureteral obstruction (UUO) as a classical model of progressive kidney injury (Eddy et al., 2012). As reported previously, MGP expression is significantly and remarkably upregulated after UUO kidney injury (Figure 5A, our unpublished data, see details in Supplementary Material). In addition to tubular epithelial cells, endothelial cells increase MGP expression after kidney injury (Figure 5B, our unpublished data, see details in Supplementary Methods). From sham-operated control and UUO-damaged kidneys, we isolated peritubular capillary endothelial cells by using FACS and compared the gene expression patterns between cells from control kidneys and those from UUO kidneys. MGP is one of the most strongly upregulated genes in endothelial cells after UUO kidney injury (Figure 5C, our unpublished data, see details in Supplementary Material). To investigate vascular protective effects of MGP against tissue injury, we subjected wild-type control mice and MGP heterozygous knockout mice (MGP^+/−^ mice) to UUO kidney injury. MGP^+/−^ mice demonstrate more severe peritubular capillary rarefaction (Figure 6A, our unpublished data, see details in Supplementary Material) and greater accumulation of α-SMA^+^ myofibroblasts than control wild-type mice (Figure 6B, our unpublished data, see details in Supplementary Material), whereas the number of infiltrating macrophages is not different between the two groups (data not shown). Peritubular capillary rarefaction is recognized to drive progression of kidney injury (Mayer, 2011; Long et al., 2012; Kida et al., 2014; Kida, 2020). These results strongly suggest that impaired MGP expression worsens vascular damage in UUO kidney injury. As we do not detect any difference in the expression of *Vegfa* transcript (mRNA of VEGF-A) between control and MGP^+/−^ mice pre- or post-injury (data not shown), VEGF-A may not be a central mechanism to explain the protective effect of MGP in this case. Figure 6C shows possible mechanisms underlying severe capillary rarefaction and intense tissue fibrosis detected in MGP knockout mice after kidney injury. In normal capillary beds, PDGFRβ^+^ kidney pericytes are closely attached to capillary endothelial cells and stabilize capillary tubes. However, endothelial cells are activated and damaged due to increased VEGF-A/VEGFR2 expression in MGP-null kidneys even without injury. In response to excessive VEGF-A signaling, endothelial cells upregulate PDGF-B expression, which triggers pericyte detachment from capillary tubes (Lin et al., 2011). MGP deficiency and kidney injury synergistically promote endothelial damage and pericyte detachment. Detached pericytes no longer stabilize capillary tubes and start to express α-SMA, leading to capillary rarefaction and impaired capillary blood flow (Kida and Duffield, 2011; Schrimpf et al., 2012; Kida et al., 2013) (Figure 6C).

## Therapeutic implications of MGP activation for vascular protection

**FIGURE 7:**
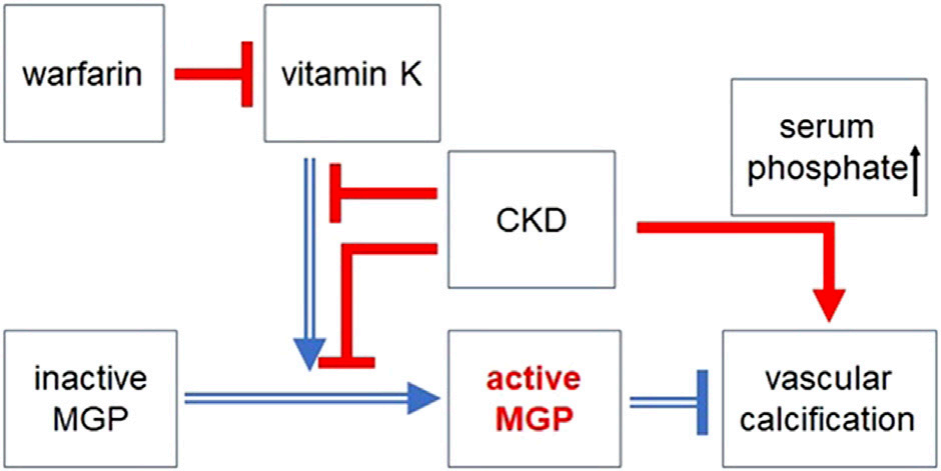
The protective effect of vitamin K dependent activation of MGP against vascular calcification. MGP is activated by γ-glutamate carboxylation in a vitamin K dependent manner. However, warfarin (the most frequently used vitamin K antagonist) impairs MGP activation *via* counteracting vitamin K. Chronic kidney disease (CKD) also blocks MGP activation. Intake of vitamin K is lowered in patients with CKD (especially in patients with hemodialysis) due to dietary restrictions and low appetite. In patients with CKD, vascular calcification is promoted by impaired MGP activation and other CKD related factors such as hyperphosphatemia, hypercalcemia, and secondary hyperparathyroidism. Open blue arrows or lines indicate beneficial effects on vascular calcification, whereas filled red arrows or lines indicate harmful effects on vascular calcification. Arrows indicate positive regulation. ⊥: negative regulation.

As impairment of MGP activity causes vascular damage, it may be important to increase the level of active MGP to protect kidneys or other organs against tissue damage. As shown in Figure 1, vitamin K controls the activation of MGP. Administration of warfarin (the most frequently used oral vitamin K antagonist) increases serum levels of inactive MGP, decreases active MGP in aortic tissues, leading to osteochondrogenic VSMCs transdifferentiation (Krűger et al., 2013). Lower vitamin K intake is associated with increased circulating levels of inactive MGP in kidney transplant recipients (Boxma et al., 2012). In animal models of CKD, vitamin K deficiency or use of warfarin exacerbates vascular calcification and increases vascular stiffness (McCabe et al., 2013). Consistent with this, warfarin use worsens arterial calcification in patients with or without CKD (Tantisattamo et al., 2015; Alappan et al., 2020). Warfarin use dramatically enhances vascular calcification in patients with end-stage renal disease (the most severe level of CKD) (Alappan et al., 2020). Independent of MGP, hyperphosphatemia, one of the major complications of CKD, directly upregulates Runx2 and induces osteochondrogenic VSMC transdifferentiation thorough suppression of Sirtuin-6, leading to vascular calcification (Li et al., 2022). Sirtuin-6 is a vascular protective protein (Kida et al., 2016). These findings indicate that vitamin K deprivation and chronic kidney injury synergistically disrupt physiological vascular structure and aggravate vascular calcification (Figure 7). However, vitamin K supplementation for 2 years does not correct coronary artery calcification, abdominal aortic calcification, nor arterial stiffness in hemodialysis patients, although plasma levels of inactive MGP (dp-ucMGP) are lowered (Levy-Schousboe et al., 2021). The process of vascular calcification is more complicated in humans than in mice. Although MGP knockout mice show severe arterial calcification early in their life, vascular calcification usually does not appear in patients with Keutel syndrome accompanying loss of function mutation in the MGP gene (Cancela et al., 2021). Activation of MGP by vitamin K monotherapy may not be enough to counteract vascular damage in patients with severe kidney injury. If possible, for patients at increased risk for harmful blood clots, warfarin would be replaced with direct oral anticoagulants (DOACs), because DOACs do not antagonize vitamin K. However, the efficacy and safety of DOACs in patients with advanced CKD are still under investigation, although some studies suggest that DOACs are not inferior to warfarin for prevention of thrombosis in patients with end-stage kidney disease and atrial fibrillation (Siontis et al., 2018).

## Conclusion

**FIGURE 8:**
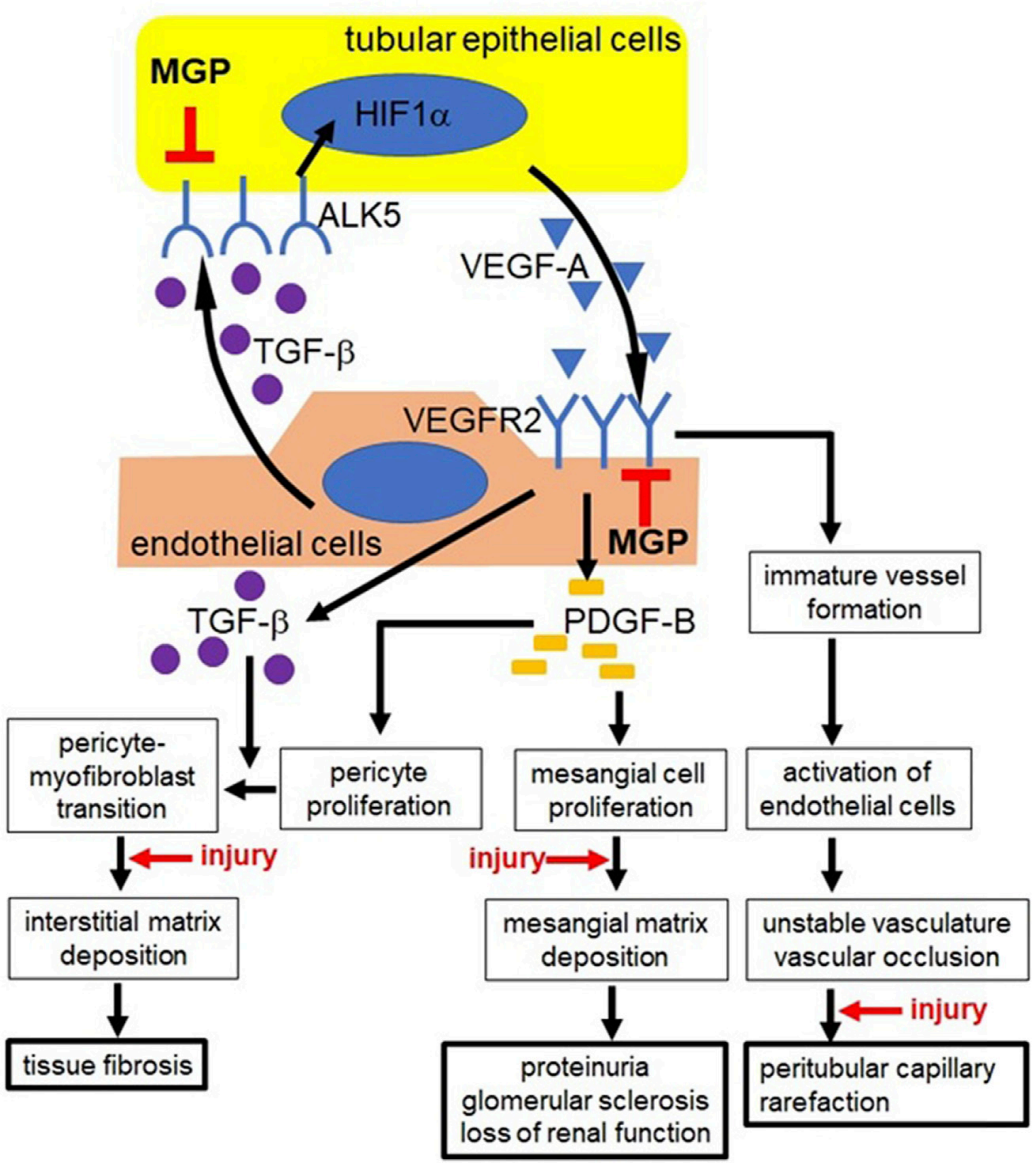
Possible mechanisms underlying increased vascular damage in MGP-deficient mice. In MGP-null kidneys, tubular epithelial cells (yellow) upregulate the expression of ALK5, leading to increased secretion of VEGF-A *via* HIF1α activation. MGP-deficient endothelial cells (red) upregulate the expression of VEGFR2. In response to increased VEGF-A, MGP-null endothelial cells synthesize and secrete excessive amount of PDGF-B and TGF-β *via* elevated VEGFR2 expression. Uncontrolled PDGF-B secretion increases proliferation of mesangial cells and interstitial pericytes. Exaggerated deposition of mesangial matrix disrupts glomerular capillary structure, causing proteinuria and glomerular sclerosis. Proliferated pericytes transdifferentiate into collagen-producing myofibroblasts *via* unrestrained expression of TGF-β, leading to tissue fibrosis. TGF-β also stimulates VEGF-A synthesis in tubular epithelial cells *via* ALK5. Intense signaling *via* VEGF-A/VEGFR2 causes abnormal angiogenesis, leading to unstable vasculature formation and vascular occlusion. These diseased events finally result in peritubular capillary regression. Kidney injury appears to enhance mesangial matrix deposition, pericyte-myofibroblast transition, formation of unstable capillaries, and capillary occlusion in MGP knockout mice. ALK5: activin receptor-like kinase receptor 5, HIF1α: hypoxia-inducible factor 1α, PDGF-B: platelet derived growth factor-B, TGF-β: transforming growth factor-β, VEGF-A: vascular endothelial growth factor-A, VEGFR2: VEGF receptor-2. ⊥: MGP inhibits excessive expression of ALK5 in tubular epithelial cells and excessive expression of VEGFR2 in endothelial cells, respectively.

Although active MGP works as a local inhibitor of vascular calcification, recent studies have revealed that MGP also controls angiogenesis during development. MGP deficiency induces abnormal angiogenesis by excessive VEGF-A/VEGFR2 signaling, leading to mild tissue damage without external injury. We discovered that impairment of MGP aggravates kidney injury due to enhancement of capillary damage, indicating that MGP has the vascular protective effect during injury. Figure 8 is a schematic showing possible mechanisms to explain how MGP deficiency induces vascular damage before and after kidney injury. However, we still do not know exactly how MGP regulates VEGF-A/VEGFR2 signaling, what upstream signaling molecules control MGP expression, and what downstream effector molecules determine outcomes of MGP signaling. Further study is necessary to answer these questions and to modify MGP signaling to treat numerous patients suffering from abnormal angiogenesis and vascular complications.
