# Supplementary material for: The vascular protective effect of matrix Gla protein during kidney injury
Source: Front Mol Med. 2022 Nov 1;2:970744. doi: 10.3389/fmmed.2022.970744 (PMC11285670; doi:10.3389/fmmed.2022.970744)
Supplement: Supplementary file 1 [file DataSheet1.zip › Supplementary Methods.pdf]

Supplementary Methods

The vascular protective effect of matrix Gla protein during kidney injury.

Yujiro Kida and Ikuyo Yamaguchi

Animals

Wild-type C57BL/6 mice were purchased from Jackson Laboratories. $MGP^{+/-}$ mice on a C57BL/6 background (Luo et al., 1997) and MGP transgenic mice ($MGP^{tg/wt}$) on a C57/BL6 background (Yao et al., 2007) were generously provided by Yucheng Yao and Kristina Boström (University of California, Los Angeles, CA, U.S.A.). We used young MGP-null mice (4 to 8 weeks old) for experiments because no MGP-null mice survived beyond 9 weeks (Luo et al., 1997). Due to a perinatal lethality of homozygous MGP transgenic mice ($MGP^{tg/tg}$), we used heterozygous MGP transgenic mice ($MGP^{tg/wt}$) for experiments (Yao et al., 2007). Genotypes were confirmed by PCR. Littermates homozygous for the wild-type allele were used as controls.

Mouse models of kidney injury

The surgical procedures were performed as previously described (Kida et al., 2013). In brief, mice were anesthetized with ketamine hydrochloride and xylazine before surgery. To create UUO injury, the left kidney was exposed through a flank incision and the left ureter was tied off at the level of the lower pole of the kidney with two 4.0 silk ties. We exposed the left kidney without ligation of the left ureter for mice in the sham group. Mice were euthanized 3 or 7 days after obstruction. All procedures complied with the guidelines established by National Research Council Guide for the Care and Use of Laboratory Animals.

Tissue preparation and histological analysis

Mouse tissue samples were prepared and studied as previously described (Kida et al., 2013). Briefly, kidneys were either snap-frozen or fixed with PLP fixative (4% paraformaldehyde [PFA]/75 mM L-lysine/10mM sodium periodate) (4°C, 2 hours), cryopreserved in 18% sucrose in PBS (4°C, overnight), and frozen. OCT-embedded (Sakura Finetek) frozen sections for immuno-fluorescence studies were blocked in 10% normal serum (Jackson ImmunoResearch) followed by incubation with a primary antibody in blocking serum (4°C, overnight). Primary antibodies used in this study included rat anti-mouse CD31 (1:500; BD Biosciences), rat anti-mouse PDGFRβ (1:200; eBioscience), FITC- or Cy3-conjugated-αSMA (1:200; Sigma-Aldrich), and rabbit anti-mouse MGP antibodies (1:100; Santa-Cruz Biotechnology). Signals of MGP and CD31 were amplified using HRP-conjugated secondary antibodies and Alexa Fluor®488-conjugated tyramide (Invitrogen). Cy3-conjugated secondary antibodies (1:200; Jackson ImmunoResearch) were used to detect signals of PDGFRβ (room temperature, 1 hour). 4',6-diamidino-2-phenylindole (DAPI) was used for visualization of nuclei. Images were captured by confocal or standard fluorescence

microscopy (Leica).

Peritubular capillary (PTC) density was analyzed as previously described (Yamaguchi et al., 2012). Briefly, images (x400) were captured from CD31-stained kidney sections. Each image was divided into 100 squares by a grid. To calculate PTC density, each square containing $CD31^+$ region resulted in a positive score. The final PTC density was represented as % of positive score.

α-SMA^+ area was analyzed as previously described (Kida et al., 2016). α-SMA^+ area was morphometrically defined and quantified by using ImageJ (National Institutes of Health, Bethesda, MD, U.S.A.).

Interstitial fibrosis was assessed using picrosirius red-stained paraffin sections and a polarized light as previously described (Kida et al., 2016).

RNA isolation and BUN measurement

Total RNA was extracted from whole kidney samples using TRIzol reagent (Invitrogen) and from isolated microvascular endothelial cells with Nucleospin RNA kit (Clonetech). Total RNA was transcribed into cDNA by iScript cDNA Synthesis kit (Bio-Rad). Quantitative reverse-transcription PCR (qRT-PCR) was performed using iQ SYBR Green Supermix kit (Bio-Rad) and iCycler (Bio-Rad). Each transcript level was normalized by glyceraldehyde-3-phosphate dehydrogenase (Gapdh) expression. Primer sequences were as follows: MGP forward 5'-GAA TCT CAC GAA AGC ATG GAG-3' and reverse 5'-CTG GAC TCT CTT TTG GGC TTT-3'; GAPDH forward 5'-AAC TTT GTG AAG CTC ATT TCC TGG TAT-3' and reverse 5'-CCT TGC TGG GCT GGG TGG T-3'.

Serum blood urea nitrogen (BUN) levels were measured in samples from MGP knockout, wild-type, and MGP transgenic mice using a kit (Teco Diagnostics).

Isolation and culture of kidney microvascular endothelial cells (MVECs)

We previously described details of this procedure (Kida et al., 2013). For preparation of kidney single cell suspension, the kidney was decapsulated, diced, and then incubated (37°C, 30 minutes) with Liberase (0.2 mg/ml; Roche) and DNase (100 IU/ml; Roche) in DMEM/F12 (Invitrogen). Digestion was curtailed by addition of DMEM/F12 including 10% fetal bovine serum (FBS). The suspension was then filtered through a cell strainer (40 μm; Fisher Scientific) to remove glomeruli and debris. After centrifugation, a cell pellet was resuspended

in cold FACS buffer (1xPBS, 0.1% BSA, 2mM EDTA, pH7.4). This kidney single suspension was sorted using FACSAria II (BD Biosciences) immediately after cells were stained with phycoerythrin (PE)-conjugated anti-CD31 (BD Biosciences), PE-Cy7-conjugated anti-CD45 (eBioscience), FITC-conjugated anti-CD11b (eBioscience) or eFluor450-conjugated anti-CD11b antibodies (eBioscience) and DAPI. We excluded DAPI$^+$ dead cells, CD11b$^+$ monocytes/macrophages, and CD45$^+$ hematopoietic cells. Then we collected a CD31$^+$ CD11b$^-$ CD45$^-$ cell population as kidney microvascular endothelial cells (MVECs).

MVECs were cultured in endothelial medium (DMEM (Invitrogen), 10% FBS, 1% penicillin/streptomycin, recombinant mouse VEGF (10 ng/ml; Peprotech), recombinant mouse basic fibroblast growth factor (20 ng/ml; Peprotech)) in 0.2% gelatin-coated plates at 37°C with 5% CO_2 and 5% O_2. All experiments were done before passage 5, because cultured MVECs were observed to start losing CD31 and VE-cadherin expression in subsequent passages.

We also tested to stain isolated MVECs for MGP and VE-cadherin. MVECs were fixed with 2% PFA, permeabilized with 0.3% Triton X-100 in PBS. Cells were stained with rat anti-mouse CD144 (VE-cadherin) antibody (1:50; BD Biosciences) or rabbit anti-mouse MGP antibody (1:100; Protein Tech) followed by incubation with Alexa Fluor Fluor®594- or Alexa Fluor®488-conjugated secondary antibody (1:200; Invitrogen), respectively.

MTS assay

The number of viable MVECs in proliferation was measured using CellTiter96 AQ_{ueous} One Solution (Promega). The AQ_{ueous} One Solution reagent contains a tetrazolium compound. MTS assay is based on the reduction of a tetrazolium compound by viable MVECs to generate a colored formazan dye that is soluble in culture media. MVECs were cultured in gelatin-coated 96-well plates in endothelium medium until 70-80% confluent. Then, cells were starved in serum-deprived medium (DMEM with 0.1% FBS, 10 ng/mL VEGF) overnight and maintained for an additional 3 days. Recombinant mouse BMP-2 (100 ng/mL; R&D Systems), recombinant mouse BMP-4 (100 ng/mL; R&D Systems), or BMP-2 and BMP-4 was added after starvation. To measure viable cells in proliferation, 20 µL of AQ_{ueous} One Solution was added and the plate was incubated at 37°C for 1 hour and read at 490 nm on a SpectraMAX 190 multi-mode microplate reader (Molecular Devices).

In-cell Western assay

This is a method for quantification of intracellular signaling in whole cells cultured in microtiter

plates. MVECs were cultured with endothelium medium in gelatin-coated 96 well plates until 70-80% confluent. MVECs were then starved in serum-deprived medium for 4 days. Cells were fixed in 4% PFA, permeabilized with cold methanol, and treated with Odyssey Blocking Buffer (LI-COR Biosciences). We incubated MVECs with primary antibodies (4°C, overnight). Primary antibodies included rabbit anti-phospho-SMAD1/5/8 (1:1000; Cell Signaling Technology) and rabbit anti-phospho-p44/42 MAPK (Erk1/2) antibodies (1:1000, Cell Signaling Technology). Primary antibodies were detected by infrared fluorescent dye-conjugated secondary antibodies. Signals of secondary antibodies were detected by an Odyssey® Infrared Imaging System (LI-COR Biosciences). Each signal was normalized by the cell number. Sapphire700 Stain (LI-COR Biosciences) was used to measure the cell number in each well.

Statistical analysis

Results were presented as mean ± standard error of the mean (SEM). Statistical significance was evaluated by Student's two-tailed *t*-test. A *P* value < 0.05 was considered significant.

References

Kida, Y., Ieronimakis, N., Schrimpf, C., Reyes, M., and Duffield, JS. (2013). EphrinB2 reverse signaling protects against capillary rarefaction and fibrosis after kidney injury. *J. Am. Soc. Nephrol.* 24:559-72. doi: 10.1681/ASN.2012080871.

Kida, Y., Zullo, JA., and Goligorsky, MS. (2016). Endothelial sirtuin 1 inactivation enhances capillary rarefaction and fibrosis following kidney injury through Notch activation. *Biochem. Biophys. Res. Commun.* 478:1074-9. doi: 10.1016/j.bbrc.2016.08.066.

Luo, G., Ducy, P., McKee, M.D., Pinero, GJ., Loyer, E., Behringer, RR., et al. (1997). Spontaneous calcification of arteries and cartilage in mice lacking matrix GLA protein. *Nature* 386:78-81. doi: 10.1038/386078a0.

Yamaguchi, I., Tchao, BN., Burger, ML., Yamada, M., Hyodo, T., Giampiero, C., et al. (2012). Vascular endothelial cadherin modulates renal interstitial fibrosis. *Nephron. Exp. Nephrol.* 120: e20-31. doi: 10.1159/000332026

Yao, Y., Nowak, S., Yochelis, A., Garfinkel, A., and Boström K. (2007). Matrix GLA protein, an inhibitory morphogen in pulmonary vascular development. *J. Biol. Chem.* 282:30131-30142. doi: 10.1074/jbc.M704297200.
